# Albumin administration prevents neurological damage and death in a mouse model of severe neonatal hyperbilirubinemia

**DOI:** 10.1038/srep16203

**Published:** 2015-11-06

**Authors:** Simone Vodret, Giulia Bortolussi, Andrea B. Schreuder, Jana Jašprová, Libor Vitek, Henkjan J. Verkade, Andrés F. Muro

**Affiliations:** 1 International Centre for Genetic Engineering and Biotechnology (ICGEB), Padriciano, 99 – 34149 – Trieste, Italy; 2 Pediatric Gastroenterology and Hepatology, Department of Pediatrics, Center for Liver, Digestive, and Metabolic Diseases, University of Groningen, Beatrix Children’s Hospital-University Medical Center, Groningen, Hanzeplein 1,9713 GZ Groningen, The Netherlands; 3 Institute of Medical Biochemistry and Laboratory Diagnostics, 1^st^ Faculty of Medicine, Charles University in Prague, U Nemocnice 2, Prague, 120 00, Czech Republic

## Abstract

Therapies to prevent severe neonatal unconjugated hyperbilirubinemia and kernicterus are phototherapy and, in unresponsive cases, exchange transfusion, which has significant morbidity and mortality risks. Neurotoxicity is caused by the fraction of unconjugated bilirubin not bound to albumin (free bilirubin, Bf). Human serum albumin (HSA) administration was suggested to increase plasma bilirubin-binding capacity. However, its clinical use is infrequent due to difficulties to address its potential preventive and curative benefits, and to the absence of reliable markers to monitor bilirubin neurotoxicity risk. We used a genetic mouse model of unconjugated hyperbilirubinemia showing severe neurological impairment and neonatal lethality. We treated mutant pups with repeated HSA administration since birth, without phototherapy application. Daily intraperitoneal HSA administration completely rescued neurological damage and lethality, depending on dosage and administration frequency. Albumin infusion increased plasma bilirubin-binding capacity, mobilizing bilirubin from tissues to plasma. This resulted in reduced plasma Bf, forebrain and cerebellum bilirubin levels. We showed that, in our experimental model, Bf is the best marker to determine the risk of developing neurological damage. These results support the potential use of albumin administration in severe acute hyperbilirubinemia conditions to prevent or treat bilirubin neurotoxicity in situations in which exchange transfusion may be required.

About 60% of healthy, term neonates, and almost all pre-term babies, will develop physiological neonatal jaundice [elevated unconjugated bilirubin (UCB) plasma levels] in the first week of life^1^,^2^, caused in most cases by a temporary delay in the uridine diphosphate glucuronosyltransferase 1A1 (*UGT1A1*) gene activation. This condition is usually considered benign^3^. However, in some babies uncontrolled unconjugated hyperbilirubinemia may develop due to other concurrent causes, such as pre-term birth, mutations in the *UGT1A1* gene (Crigler-Najjar syndrome), hemolytic conditions (such as glucose-6-phoshate dehydrogenase deficiency, other genetic disorders affecting hemoglobin or the erythrocyte membrane, or immune mediated hemolysis), sepsis, or other unknown stressors, resulting in acute bilirubin encephalopathy (kernicterus) and, eventually, death^2^,^3^,^4^. The incidence of kernicterus is about 0.4 to 2.7 per 100,000 live births^4^,^5^, raising to about 1.8 per 1000 live births considering preterm infants born with less than 30 weeks of gestational age^6^. Yet, it is significantly more frequent in underdeveloped and developing countries^5^,^7^,^8^,^9^,^10^, being a “silent” cause of significant neonatal morbidity and mortality. In fact, it is ranked as one of the three top causes of death among African newborns^9^,^11^,^12^,^13^.

Jaundice is normally treated with phototherapy (PT), which for most patients has sufficient efficacy and convenience, with high safety and low costs. However, jaundiced infants who fail to respond to PT, or are severely hyperbilirubinemic upon first presentation, are treated with a more invasive and inherently more dangerous alternative, such as exchange transfusion (ET). ET is implemented only in specialized centers and carries a significant risk of morbidity and mortality from vascular accidents, cardiac complications, biochemical and haematological disturbances^2^,^14^. The overall mortality rate from the procedure, having high variability among the different centers, is normally quoted as being 0.3–0.7%, although it may reach up to 17% in developing countries^15^. Adverse events may amount up to 36%, including catheter-related complications, sepsis, thrombocytopenia, and hypocalcemia^9^,^16^,^17^,^18^,^19^,^20^. Therefore, the development of alternative strategies to reduce the risk of bilirubin encephalopathy in a rapid and efficacious manner is a clinical need.

At a physiological pH bilirubin is poorly water-soluble and therefore needs to be metabolized in the liver to allow its disposal. Due to the high affinity of albumin for unconjugated bilirubin^21^, UCB is transported via the circulation to the liver bound to albumin^22^. Neurological damage is produced by the small fraction of UCB not bound to albumin (free bilirubin, Bf). Bf, normally present at levels lower than 0.1% of total bilirubin in plasma^23^, is capable of crossing the blood-brain-barrier and disrupting several essential cellular functions, resulting in neuronal cell death^24^,^25^. Therefore, a possible strategy to avoid bilirubin accumulation in the brain could be to increase the bilirubin-binding capacity within the intravascular compartment by albumin supplementation. Administration of albumin, prior to ET, has been performed in the pediatric clinic to increase the efficacy of this procedure^26^,^27^,^28^,^29^,^30^,^31^,^32^. Nonetheless, the therapeutic use of albumin is not routinely used to treat severe neonatal jaundice due to the absence of strong experimental support^14^.

Pre-clinical studies performed by our group in the Gunn rat model of hyperbilirubinemia demonstrated the short term efficacy of a single albumin infusion in lowering plasma Bf, brain bilirubin levels and preventing brainstem evoked potential alterations^33^,^34^. However, the mild phenotype of the Gunn rats did not allow the study of the long-term effect or the therapeutic efficacy of the treatment to save lives was determined. Hyperbilirubinemia in Gunn rats is normally not associated with kernicterus, and they develop acute central nervous system dysfunction, and eventually irreversible brain damage, only when further challenged by administration of bilirubin-albumin displacers (e.g. sulphonamides) or of erythrocyte-lysing agents such as phenylhydrazine^35^.

In the present work, we performed one crucial step forward: we tested the long-term benefits of albumin administration in a more severe and clinically relevant lethal mouse model of neonatal hyperbilirubinemia^36^,^37^, without the application of the standard phototherapy treatment. These mice lack bilirubin-conjugation activity and develop severe hyperbilirubinemia soon after birth^36^,^37^, closely mimicking the human pathology. Mutant mice show important cerebellar defects, neuronal cell death and die shortly after birth due to bilirubin neurotoxicity^36^.

We show here that repeated albumin administration, without PT application, prevents bilirubin-induced neurological damage and lethality. We demonstrate that the albumin dosage and administration frequency are determinants for preventing bilirubin neurotoxicity. The present work also provides evidence that in our experimental model Bf is the best predictor of neurological damage induced by bilirubin, among the most commonly used clinical markers.

## Results

### Daily human serum albumin (HSA) administration increases survival of the mutant mice

**Figure 1:**
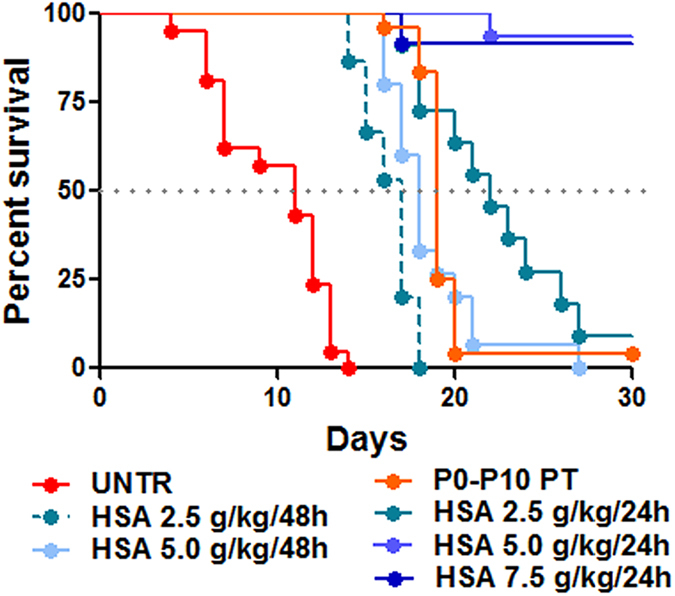
Administration of HSA increase survival of mutant mice. Kaplan-Meier survival curve of FVB/NJ *Ugt1* mutant mice. Mutant mice were treated with IP injections of albumin (2.5, 5.0 and 7.5 g/kg) from P2 to P20 every 24 h or 48 h, as indicated. The line color/type indicates the different treatments (red line, untreated mutant mice; orange line, mutant mice treated with phototherapy from P0 to P10; other lines, HSA treatments). *p* < 0.0001, Log-rank (Mantel-Cox) test. The number of animals per treatment is as follows: UNTR (n = 21), P0-P10 PT (n = 24), HSA 2.5 g/kg/48 h (n = 15), HSA 2.5 g/kg/24 h (n = 11), HSA 5.0 g/kg/48 h (n = 15), HSA 5.0 g/kg/24 h (n = 15), HSA 7.5 g/kg/24 h (n = 12) UNTR, untreated mice; HSA, human serum albumin; PT, phototherapy; P, post-natal day.

Figure 1 shows the effects of intraperitoneal (i.p.) administration of human serum albumin (HSA) on the survival of mice, at different time intervals and dosages. Administration of either 2.5 g/kg or 5.0 g/kg HSA doses every 48 h (HSA 2.5 g/kg/48 h and HSA 5.0 g/kg/48 h, respectively) effectively delayed mortality of mutant mice (50% survival at post-natal day 17 and 18, P17 and P18, respectively, Fig. 1), but all mutant mice died before day 27 after birth. HSA administration of 2.5 g/kg every 24 h (HSA 2.5 g/kg/24 h) resulted in higher survival of mice (50% mortality at P22), with one out of 11 treated mice surviving beyond 30 days. Finally, daily treatment with HSA 5.0 g/kg (HSA 5.0 g/kg/24 h) or 7.5 g/kg (HSA 7.5 g/kg/24 h) resulted in about 95% survival of mice beyond 30 days (Fig. 1). These results strongly underscored that HSA administration reduces mortality in this mouse model in a dose and time-dependent manner.

To determine potential side effects of albumin treatment, we analyzed several parameters. We monitored HSA-treated animals by weighing them daily since birth, and we did not observe any evident alteration in their weight curve, routine behavior and general aspect, indicating that mice tolerated well the treatment, even at the highest dosages (Supplementary Fig. 1A). Moreover, we determined ALT and AST at P15 in animals receiving daily HSA administration to evaluate possible liver damage (Supplementary Fig. 1B–C). We observed that plasma alanine aminotransferase (ALT) and aspartate aminotransferase (AST) activities were not significantly different from wild type (WT) and mutant (MUT) uninjected controls, indicating the absence of liver damage by HSA daily treatment.

**Figure 2:**
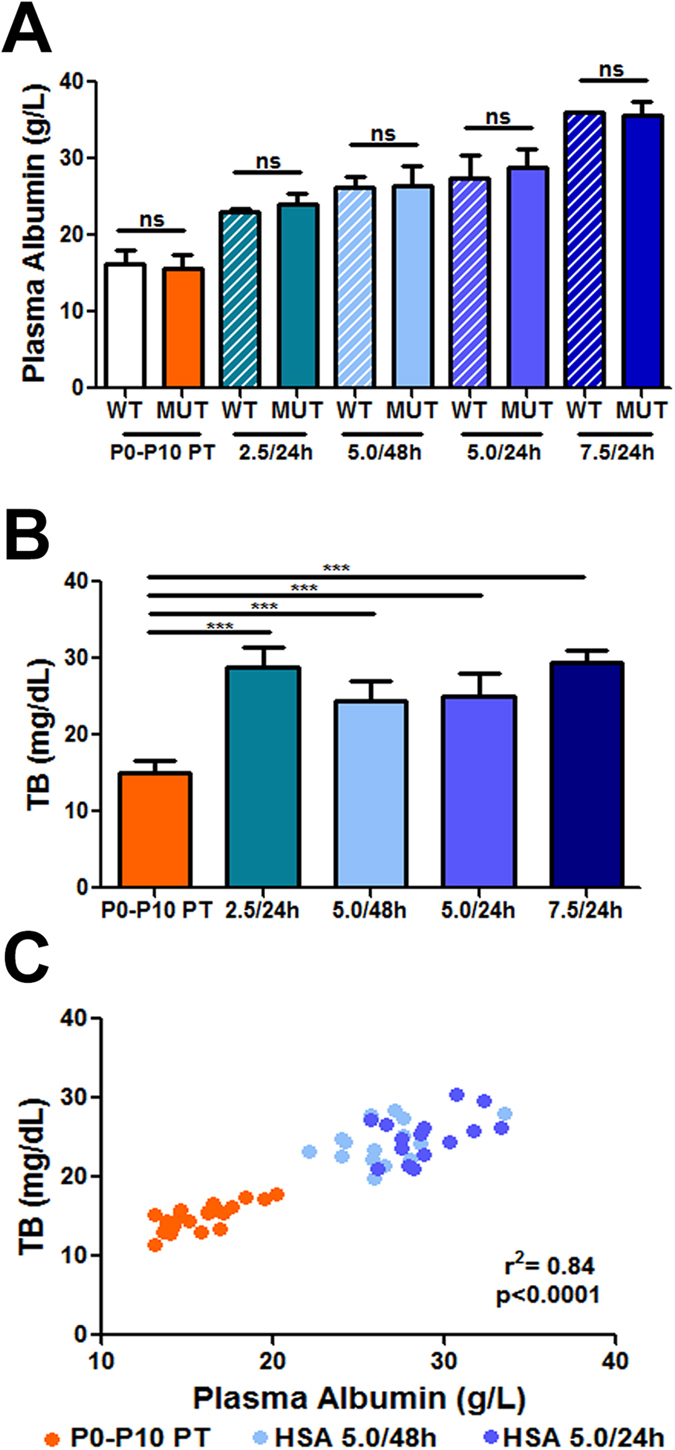
Dose-dependent effect of albumin administration on plasma values. (**A**) Plasma albumin levels in untreated and HSA injected mutant and WT mice at P15. Values represent mean ± SD (g/L). *t-*test, not significant. P0-P10 PT (WT = 20, MUT = 20), HSA 2.5 g/kg/24 h (WT = 5, MUT = 7), HSA 5.0 g/kg/48 h (WT = 4, MUT = 15), HSA 5.0 g/kg/24 h (WT = 5, MUT = 15), HSA 7.5 g/kg/24 h (WT = 1, MUT = 3); **(B)** Total plasma bilirubin levels in mutant mice at P15. Values represent mean ± SD (mg/dL). One-way ANOVA test, ***p < 0.001. P0-P10 PT (n = 20), HSA 2.5 g/kg/24 h (n = 7), HSA 5.0 g/kg/48 h (n = 15), HSA 5.0 g/kg/24 h (n = 15), HSA 7.5 g/kg/24 h (n = 3); (**C**) Correlation test between plasma albumin and total bilirubin (TB) in P0-P10 PT, HSA 5.0 g/kg/48 h and HSA 5.0 g/kg/24 h-treated mutant mice. Each dot corresponds to a single animal. Correlation test, Pearson coefficient WT, wild-type; MUT, mutant; HSA, human serum albumin; PT, phototherapy; P, post-natal day; TB, total bilirubin; ns, not significative.

Then, we determined plasma albumin and total bilirubin concentration in treated mice at P15, 24 h after the P14 HSA injection in all treated groups (Fig. 2). Since untreated mutant mice did not survive longer than P15 [36 and Fig. 1, red line], we therefore selected as control group an experimental condition that allows the animals to survive longer, by temporarily treating them with PT [12 h/day since birth up to P10 (P0-P10 PT), and then transferring the mice to normal light conditions). Bilirubin rapidly raises after discontinuation of the PT treatment, being at P15 not any longer affected by the PT treatment^36^. Under these experimental conditions, only about ~5% of P0-P10 PT – treated mutant mice survived after P30 (Fig. 1, orange line). Albumin treatment, as expected, increased plasma albumin concentrations in a dose-dependent manner, up to a maximum increase of about two-fold (Fig. 2A). No differences in basal albumin concentration between P0-P10 PT mutant and WT mice were observed, or between WT- and HSA-treated mutant mice, within each treatment (Fig. 2A and Table 1).

Albumin treatment increased plasma bilirubin concentration in HSA-treated mutant mice, up to two-fold higher than in controls (HSA 7.5 g/kg/24 h vs. P0-P10 PT; 29.4 mg/dL and 14.95 mg/dL, respectively, *t*-test, *p* < 0.001, Fig. 2B). Plasma bilirubin levels of HSA-treated WT mice were similar to untreated WT mice, in the range of 0.5 mg/dL (Table 1).

Daily administration of 5.0 g/kg albumin was the minimal dosage resulting in virtually complete survival of mutant mice, while the 5.0 g/kg dose administered every 48 h resulted in death of all animals. This apparent critical dosage scheme led us to compare the relevant parameters at P15.

**Figure 3:**
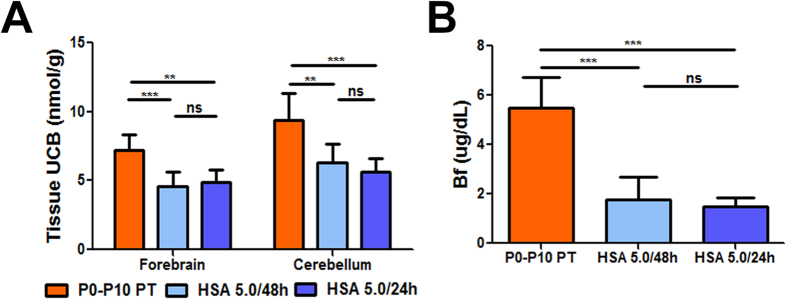
Effect of albumin supplementation on tissue bilirubin-binding and Bf. (**A**) Brain UCB levels (forebrain and cerebellum) in P0-P10 PT (n = 8), HSA 5.0 g/kg/48 h (n = 6) and HSA 5.0 g/kg/24 h (n = 7)-treated mutant mice at P15. Values represent mean ± SD (nmol/mg). One-way ANOVA test, **p < 0.01, ***p < 0.001. (**B**) Free bilirubin (Bf) analysis in plasma in P0-P10 PT (n = 19), HSA 5.0 g/kg/48 h (n = 9) and HSA 5.0 g/kg/24 h (n = 8) mutant mice at P15. Values represent mean ± SD (μg/dL). One-way ANOVA test, ***p < 0.001 HSA, human serum albumin; PT, phototherapy; P, post-natal day; UCB, unconjugated bilirubin; ns, not significative.

Figure 2C shows the strong correlation between plasma albumin and plasma total bilirubin (TB) concentration values in the HSA 5.0 g/kg/24 h, HSA 5.0 g/kg/48 h and P0-P10 PT groups (Fig. 2C; r^2^ = 0.84, p < 0.0001, Correlation test, Pearson coefficient). Since the cause of neurotoxicity is the bilirubin accumulated in the tissue, we then determined the levels of tissue bilirubin in forebrain and cerebellum, which were 30–40% lower in both HSA 5.0 g/kg/24 h and HSA 5.0 g/kg/48 h groups, compared with control mutant mice (P0-P10 PT group) (Fig. 3A). Interestingly, there was no difference between the 24 h and the 48 h-treated groups. Indeed, determination of plasma Bf at P15 showed that albumin supplementation significantly reduced the free fraction of bilirubin capable of causing damage (Fig. 3B, p < 0.001, ANOVA). In fact, Bf in HSA-treated animals was about 33% of the P0-P10 PT control group, but there was no difference between the two HSA-treated groups, which received different frequencies of injection.

**Figure 4:**
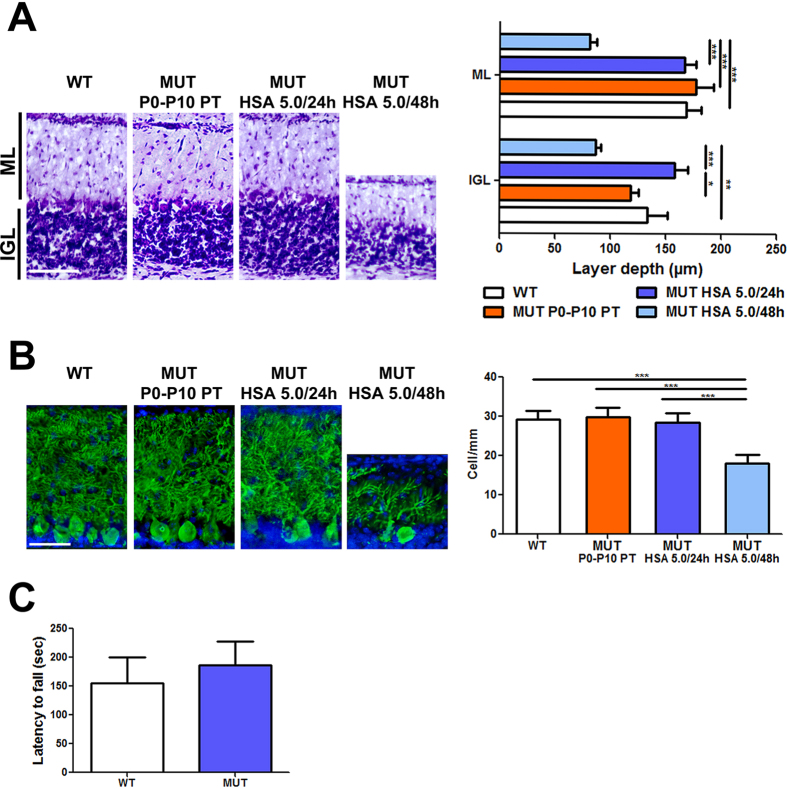
Neurological assessment of albumin treatment. WT, P0-P10 PT, HSA 5.0/48h and HSA 5.0/24h-treated mutant mice cerebellar analysis at P15. (**A**) Left panel, Nissl staining of cerebellar internal granular layer and molecular layer. Right panel, layer depth quantification. Scale bar 100  μm. Values represent mean ± SD (μm). One-way ANOVA test, *p < 0.05, **p < 0.01, ***p < 0.001. WT(n = 7), P0-P10 PT (n = 4), HSA 5.0 g/kg/48 h (n = 3), HSA 5.0 g/kg/24 h (n = 3); (**B**) Left panel, representative fluorescent immunohistochemistry. PCs were stained with anti-calbindin1 antibody (green) and nuclei with Hoecsht stain (blue). Right panel, quantification of PCs is represented in the bar. Scale bar 50 μm. Values represent mean ± SD (cell/mm). One-way ANOVA test, ***P < 0.001. WT (n = 9), P0-P10 PT (n = 4), HSA 5.0 g/kg/48 h (n = 5), HSA 5.0 g/kg/24 h (n = 5); (**C**) Motor coordination of WT (n = 24) and rescued treated mutant mice (20) on rotarod at 1 month of age. Values represent mean ± SD (s). *t-*test, not significant. ML, molecular layer, IGL, internal granular layer, PC, Purkinje cell HSA, human serum albumin; WT, wild-type; MUT, mutant; PT, phototherapy; P, post-natal day; UCB, unconjugated bilirubin; ns, not significative.

Next, we performed histological analysis of the cerebellum, the most affected brain region^36^,^37^, at P15. Mutant mice treated with HSA 5.0  g/kg/24  h showed cerebellar layers similar to WT, while we observed an important reduction of the layers’ depth in the HSA 5.0  g/kg/48 h group (50% reduction in the internal granular layer and molecular layer, Fig. 4A). As previously observed, the P0-P10 PT treatment was sufficient to prevent major abnormalities in cerebellar development (Fig. 4A,B)^36^. Calbindin-specific staining of Purkinje cells (PCs) showed normal PCs density and dendritic arborization in both P0-P10 PT and HSA 5.0 g/kg/24 h animals, but a 40–50% reduction in the number of PC and their dendritic arborization in the HSA 5.0 g/kg/48 h group (Fig. 4B). Importantly, HSA-rescued animals did not show any obvious motor-coordination impairment compared to WT littermates, as assessed by the accelerating rotarod test at 1 month of age (Fig. 4C). These results were in line with the histological analysis, confirming that repeated HSA administration prevents bilirubin-induced neurological damage.

### Frequency of HSA administration is crucial to prevent bilirubin accumulation in brain and bilirubin-induced neurological damage

Since the differences in histology and survival between the 5.0 g/kg/24 h and 5.0 g/kg/48 h-treated mutant mice were not supported by any difference in the parameters determined at P15 (TB, Bf and tissue bilirubin), we reasoned that this could be related to the timing of albumin administration (both groups had received the last HSA dose 24 h before, at P14).

**Figure 5:**
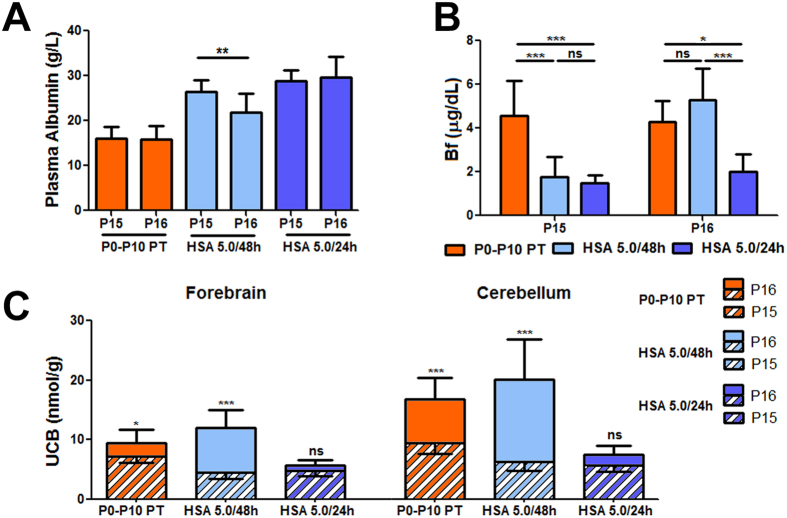
Plasma albumin, Bf and brain UCB at P16. Increment of Bf and tissue UCB in the HSA 5.0/48 h group 48 h after the last albumin administration. Comparison between P15 and P16 in P0-P10 PT, HSA 5.0 g/kg/48 h and HSA 5.0 g/kg/24 h-treated mutant mice. (**A**) Plasma albumin levels decrease in HSA 5.0 g/kg/48 h treated mutant mice. Values represent mean ± SD (g/L). *t-*test, *p < 0.05. P0-P10 PT (n = 6), HSA 5.0 g/kg/48 h (n = 14), HSA 5.0 g/kg/24 h (n = 7); (**B**) Bf plasma levels at P15 and P16. Values represent mean ± SD (μg/dL). One-way ANOVA test, *p < 0.05, ***p < 0.001. P0-P10 PT (n = 6), HSA 5.0 g/kg/48 h (n = 7), HSA 5.0 g/kg/24 h (n = 7); (**C**) Brain UCB content. The stripped bars represent the amount of UCB at P15, while the full colored bars represent the increment of UCB levels from P15 to P16 (the whole bars, stripped plus full colored, represent UCB levels at P16). *t-*test, *p < 0.05, ***p < 0.001. P0-P10 PT (n = 6), HSA 5.0 g/kg/48 h (n = 8), HSA 5.0 g/kg/24 h (n = 6). The number of animals analyzed at P15 is indicated in the legend to Figs 2 and 3 HSA, human serum albumin; PT, phototherapy; P, post-natal day; Bf, free bilirubin; ns, not significative.

Therefore, we investigated the second 24 h of the HSA administration in the 5.0 g/kg/48 h group. At this time point (P16), the HSA 5.0 g/kg/48 h group received the last HSA injection 48 h before the analysis (at P14). Conversely, only 24 h passed for the HSA 5.0 g/kg/24 h-treated animals that received the last injection at P15 (Fig. 5).

Plasma albumin determination showed a significant reduction of about 25% between P15 and P16 in the HSA 5.0 g/kg/48 h group (Fig. 5A, p < 0.001, *t-*test). On the contrary, no differences were observed for the 5.0 g/kg/24 h and P0-P10 PT groups at those time points.

Plasma Bf values 48 h after the last injection triplicated (from P15 to P16) in the HSA 5.0 g/kg/48 h group (Fig. 5B), reaching values of the untreated control group. In contrast, Bf levels remained steady and low in the HSA 5.0 g/kg/24 h group.

Determination of tissue bilirubin showed a significant increase in the P0-P10 PT control group, both in the forebrain and in cerebellum, associated with the physiological raise in TB plasma levels between P15 and P16 (Supplementary Fig. 2; Fig. 5C). In addition, in the 5.0 g/kg/48 h-treated group tissue bilirubin rose significantly as a consequence of the concomitant increase of Bf in plasma (Fig. 5B). In contrast, there was no variation in tissue UCB in the HSA 5.0 g/kg/24 h group, strongly indicating that daily HSA infusions can keep under safe therapeutic levels tissue UCB, avoiding bilirubin toxicity and neurological damage.

### Predictive markers of bilirubin-induced neurological damage

**Figure 6:**
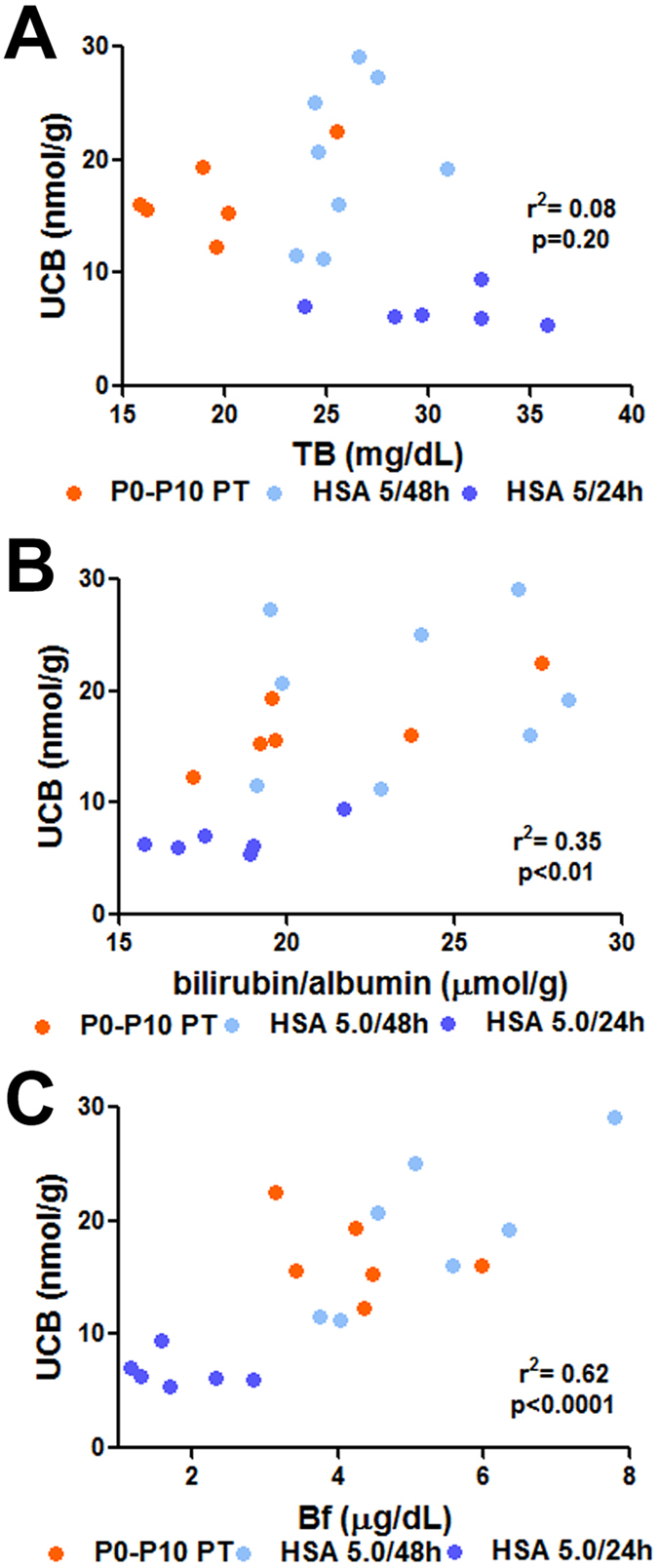
Plasma markers of bilirubin neurotoxicity. The different parameters routinely used in the clinics to monitor hypebilirubinemia were analyzed at P16 as a function of tissue bilirubin in the cerebellum (nmol/g), to determine the best indicator of cerebellar bilirubin neurotoxicity. (**A**) Total bilirubin, TB (mg/dL); (**B**) Bilirubin/albumin (B/A) ratio (μmol/g); and (**C**) Unconjugated free (unbound) bilirubin, Bf (μg/dL). Each dot represents a single animal. P0-P10 PT (n = 6), HSA 5.0 g/kg/48 h (n = 8), and HSA 5.0 g/kg/24 h (n = 6) HSA, human serum albumin; PT, phototherapy; P, post-natal day; UCB, unconjugated bilirubin; Bf, free bilirubin; ns, not significative.

Since cerebellum is the most affected brain region, we selected cerebellar bilirubin content as an estimator of neurological damage. We evaluated which parameter better predicted neurological damage and survival by plotting individual data of plasma TB concentration, B/A ratio and Bf as a function of tissue bilirubin levels at P16 (Fig. 6).

We observed that neither plasma TB nor bilirubin/albumin B/A ratio correlated with cerebellar bilirubin content, being the distribution of both populations overlapping with that of the HSA 5.0 g/kg/24 h group (Fig. 6A, B; r^2^ = 0.08 and 0.35, respectively; *p* = 0.2, *p* < 0.01, respectively, Correlation test, Pearson coefficient). In contrast, plasma Bf values showed a more clear separation between both treatments. The Bf of all HSA 5.0 g/kg/24 h animals were clearly distinct from the Bf values of the two other groups (HSA 5.0 g/kg/48 h and P0-P10 PT) (Fig. 6C, r^2^ = 0.62, *p* < 0.0001, Correlation test, Pearson coefficient), indicating that Bf better predicts bilirubin neurotoxicity in our experimental model, as determined at P16.

## Discussion

Aiming to decrease bilirubin toxicity in the brain, our group recently demonstrated that the combination of a single HSA infusion with PT was very effective in lowering plasma free unconjugated bilirubin, brain bilirubin levels and preventing brainstem evoked potential alterations, using the Gunn rat^33^,^34^, a non-lethal model of hyperbilirubinemia. In the present work, we made one crucial step forward by using a mouse model for hyperbilirubinemia showing early neonatal lethality^36^,^37^ and treating mutant pups with repeated HSA infusions since birth, without the application of PT. In this way, we evaluated the effectiveness of the long-term sustained increase in bilirubin-binding capacity in plasma to prevent neonatal neurological damage and death.

We demonstrated that daily HSA administration during postnatal development was necessary and sufficient to rescue neurological damage and lethality. Rescued mutant mice showed normal motor coordination abilities, no histological abnormalities in the cerebellum and normal albumin levels at follow up (at postnatal day 30).

Moreover, HSA administration clearly increased bilirubin-binding capacity in plasma, evident by the drop in Bf levels and by the impressive increase in plasma TB in treated animals, indicating that that the therapeutic efficacy of albumin was thus not mediated by a total bilirubin lowering effect, but by the reduction of Bf concentration.

Our data strengthen the concept that TB increase in plasma results from the mobilization of bilirubin from tissues. Most importantly, despite the extreme hyperbilirubinemia in the plasma compartment, HSA-treated animals survived without any adverse effect, as a consequence of having much lower tissue UCB levels.

Importantly, frequency of administration was critical to determine survival or death of mutant mice. In fact, daily administration maintained therapeutic albumin levels in plasma, and guaranteed normal brain development and survival. In contrast, HSA administration every 48 h resulted in a critical increase in tissue UCB leading to important abnormalities in cerebellar development, and death. This increase in plasma and tissue UCB levels was associated with an important decrease in plasma albumin levels 48 h after the last administration (HSA 5.0 g/kg/48 h at P16). It remains unclear the reasons of the rapid decrease in plasma albumin concentration 48 h after administration since the reported life-time of monomeric albumin in humans is in the range of 28–36 days^38^. However, our finding is in line with that observed in analbuminemic patients and adult Gunn rats infused with HSA^33^,^39^,^40^, where plasma albumin levels increased immediately after infusion, but substantially decreased 24 h post-treatment. Moreover, it was shown that albumin half-life in mice infused with mouse albumin was 35 h^41^ while that of mice infused with HSA was 21 h^42^. We speculate that it could be related to its distribution in the extravascular space of other body compartments, resulting in a reduction in the plasma levels, and/or to species-specific differences leading to faster albumin degradation^42^,^43^.

In line with our observations are Hosono *et al.*^28^ data, showing promising results in infants treated with a single infusion of albumin at the beginning of the PT treatment, resulting in a decrease of auditory brainstem response abnormalities. We cannot exclude that lower doses of albumin could have a similar or higher therapeutic effect if administered even more frequently (i.e., twice a day).

In the present work, the dosage scheme of 5.0 g/kg daily was the minimal one able to rescue mortality. The usual dose administered in neonates is lower than the one used in this study (about 1.0 g/kg, administered i.v.)^28^,^30^, even though in some specific cases doses up to 4.5 g/kg were used without evident adverse effects^31^,^32^,^44^. Here, we did not observe any obvious secondary effects, even when animals were treated with the highest dose (7.5 g/kg every 24 h). Although effective, dose comparison between mice and neonates is not trivial, especially when different administrations routes are applied. In fact, due to the small size of the mouse neonates (about 2 g at P2), we adopted intraperitoneal injection, a procedure that results in a slower availability of administered HSA in the intravascular compartment, compared to intravenous administration, being routinely used in newborns. Therefore, we can speculate that the more frequent HSA administration and the use of the i.v. route in neonates may require the administration of lower doses than the ones used here. Thus, these procedures, in combination with the application of intensive phototherapy, may limit the concerns raised by the high HSA doses administered to the present model. However, the potential benefits of HSA application have to be more deeply investigated in human jaundiced babies, especially in preterm infants with very low weight at birth, being the most susceptible to bilirubin neurotoxicity.

Due to the elicited increase in plasma bilirubin, albumin administration to reduce bilirubin-induced neurological damage invalidates the use of plasma TB as an indicator of the overall risk of bilirubin neurotoxicity. Clinical indications in patients suggest that TB levels over a threshold value of ~20 mg/dL, are poor discriminators of the individual risk of developing brain damage^45^,^46^. Hence, other more specific indicators are a clinical need.

It has been proposed that the ratio of bilirubin to albumin or B/A^47^ could be a valid parameter to approximate the risk of bilirubin neurotoxicity in neonates. B/A was endorsed by the American Academy of Pediatrics^2^ and routinely used to determine the threshold for ET^14^,^47^. Our data indicate that B/A ratio is a poor indicator of neurological damage and death in our mouse model. In fact, the B/A ratio, similarly to the TB values, was not able to accurately predict the outcome of the different groups of mice used here. However, this study differs markedly from the application of the B/A ratio to predict neurotoxicity risk in human neonates, in which bilirubin levels are elevated in the context of significant hypoalbuminemia. In fact, we artificially increased the plasma albumin concentration in the pups by repeated albumin administration. In recent results obtained in the BARTrial, the B/A ratio was found to be similarly effective as TSB in the management of hyperbilirubinemia in preterm infants to prevent neurodevelopmental damage^48^.

The concentrations in plasma of unbound unconjugated bilirubin, Bf, correlated strongly with the beneficial treatment effects. Accordingly, we provided here compelling supportive evidence that the accurate determination of the free pool of bilirubin (Bf) concentration in plasma could act as an indicator of the risk of neurological damage.

The potential use of Bf in human patients, as a precise parameter to predict bilirubin-induced neurological damage and kernicterus is supported by various studies^25^,^46^,^49^,^50^,^51^. These authors showed that defects in auditory brainstem response in a newborn population, caused by bilirubin-induced neurotoxicity, correlates with Bf rather than total plasma bilirubin. In another study performed in extremely low birth weight infants, Bf was associated with death or adverse neurodevelopmental outcomes and was a better predictor than TB, since its correlation was independent of clinical status^52^. Consistently, a recent study shows that high Bf predicts kernicterus in extremely low birth weight Japanese infants^51^.

Based on our data in mice, albumin administration can clearly confer protection to bilirubin neurotoxicity and save lives. HSA beneficial effects were even obtained with the exclusive use of albumin, i.e as mono-therapy. It is reasonable to assume that similar added value is possible upon co-treatment with PT. Thus, this procedure could be used in therapy resistant hyperbilirubinemia or (imminent) kernicterus. It may also be helpful to acute patients when the concomitant implementation of ET is not easily or rapidly possible, either by patient related factors or infrastructure. We have previously shown in a preclinical rat model that PT treatment enhances the effect of albumin administration^33^,^34^. Indeed, we propose that the therapeutic synergy of intensive PT and frequent albumin administration, in combination with the constant monitoring of free unbound bilirubin (Bf), may result in the most effective and feasible procedure to be applied to such jaundiced neonates.

ET is normally applied in the context of the clinical scenarios of infants presenting with hazardous hyperbilirubinemia or who fail to respond to PT^2^,^14^, with high risk of mortality and adverse events^17^,^18^,^19^,^20^. It is important to highlight that the procedure described here, which could be an efficient adjuvant treatment to intensive phototherapy, is at the reach of most neonatal care units in a quick and secure manner, and should present almost no concerns regarding its safety profile.

In conclusion, the results presented here supports the potential use of albumin infusions in severe acute neonatal hyperbilirubinemia and in Crigler-Najjar patients, to limit/avoid bilirubin neurotoxicity in situations in which ET may be required. We also demonstrated that the dosage and administration frequency are critical parameters for its efficacy. Finally, our data provide evidence that in this lethal mouse model, plasma Bf concentration is the best marker to predict the risk of bilirubin-induced brain damage. Randomized clinical trials comparing the efficacy of albumin therapy versus other modalities are warranted to confirm our experimental data.

## Methods

### Animals

Mice were housed and handled according to institutional guidelines, and experimental procedures approved by the ICGEB board, with full respect to the EU Directive 2010/63/EU for animal experimentation. *Ugt1* mutant mice in the FVB/NJ background have been generated previously^36^,^37^. Homozygous mutant animals were obtained from heterozygous matings. WT littermates were used as control. Average litters were of 9–10 pups. No loss of pups was observed. Animals used in this study were at least 99.8% FVB/NJ genetic background, obtained after more than ten backcrosses with wild type FVB/NJ mice. Mice were kept in a temperature-controlled environment with 12/12 h light/dark cycle. They received a standard chow diet and water *ad libitum*.

### Phototherapy treatment

Phototherapy treatment was performed as previously described^36^. Animals of the P0-P10PT control group were exposed to blue fluorescent light (20 μW/cm^2^/nm, Philips TL 20W/52 lamps; Philips, Amsterdam, The Netherlands) for 12 hours/day since birth up to postnatal day 10, and then maintained under normal light conditions.

### Albumin treatment

Newborn mice were intraperitoneally (i.p.) injected with HSA (Albuman®; solution for infusion, 200 g/L, fatty acid free) purchased from Sanquin (Amsterdam, The Netherlands). WT (injected control group) and mutant mice were injected from postnatal day 2 (P2) up to postnatal day 20 (P20), with 2.5 g/kg/48 h (n = 15), 5.0 g/kg/48 h (n = 15), 2.5 g/kg/24 h (n = 11), 5 g/kg/24 h (n = 15) and 7.5 g/kg/24 h (n = 12) and survival was monitored. Animals treated every 48h received HSA administration at P2, P4, P6, P8, P10, P12, P14, P16, P18 and P20, while the 24 h group every 24 h, starting at P2 till P20.

### Biochemical analyses of plasma samples

Blood samples were collected at different time points in mutant and WT littermates by cardiac puncture in EDTA-collecting tubes, at the moment of sacrificing the animals, as previously described^36^. Total bilirubin (TB), Bf, and albumin were determined as described^33^,^53^,^54^.

### Tissue bilirubin analysis

Tissues for bilirubin content determination were collected and analyzed as previously described^53^,^55^.

### Rotarod analysis

At 1 month of age, the coordination and balance ability of mutant and WT mice were tested on a rotating cylinder with an accelerating apparatus, as previously described^37^.

### Brain histology

Histological and immunofluorescence analysis of forebrain and cerebellum samples was performed as previously described^36^,^37^. The study was performed in a double-blind fashion: the genotype of the animals and the treatment were unknown to the surgeon, while a different investigator analyzed the data. Measurements were averaged for each animal.

### Statistics

The Prism package (GraphPad Software, La Jolla, CA) was used to analyze the data. Results are expressed as mean ± s.d. Values of *p* < 0.05 were considered statistically significant. Depending on the experimental design, Student’s *t*-test or one-way ANOVA, with Bonferroni’s post-hoc comparison tests were used, as indicated in the legends to the figures and text. Correlation analyses were done using the Pearson coefficient to assess the linearity between two variables and calculate two-tailed p value (95% of confidence interval).

## Additional Information

**How to cite this article**: Vodret, S. *et al.* Albumin administration prevents neurological damage and death in a mouse model of severe neonatal hyperbilirubinemia.. *Sci. Rep.* **5**, 16203; doi: 10.1038/srep16203 (2015).

## Supplementary Material

Supplementary Information

## Figures and Tables

**Figure 7:**
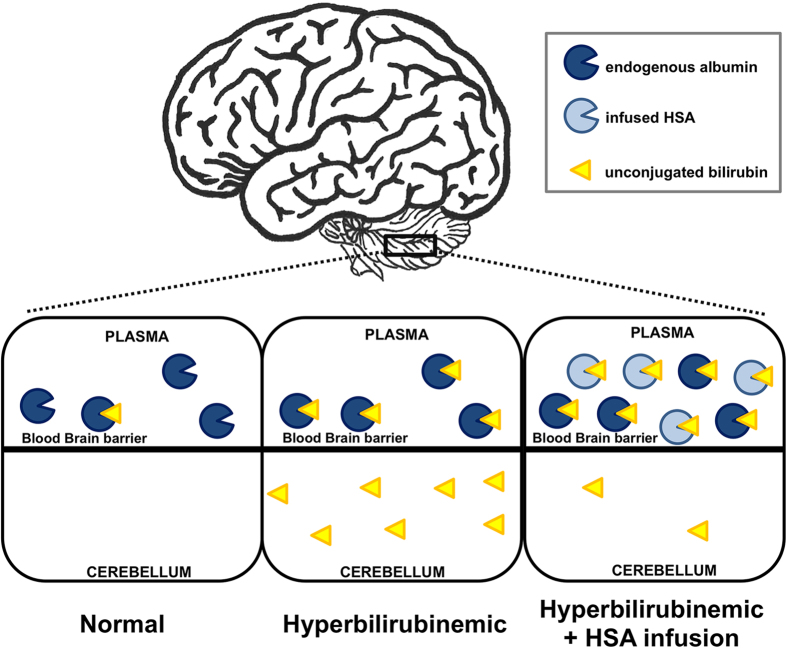
Model of bilirubin mobilization by HSA administration. In normal conditions, the bilirubin-binding capacity provided by albumin exceeds the amount of UCB (left). In severe hyperbilirubinemic conditions, UCB outnumbers the albumin-binding capacity, and the excess of UCB (free bilirubin) solubilizes in lipid-rich tissues, such as the brain and cerebellum (center), resulting in neurological damage. When plasma bilirubin-binding capacity is artificially increased by HSA administration, bilirubin is mobilized from tissues to the plasma compartment, resulting in safe levels of tissue UCB (right) and increased plasma UCB levels. Thus, bilirubin mobilization prevents neurological damage and rescues lethality in HSA-treated mutant mice UCB, unconjugated bilirubin; HSA, human serum albumin.

**Table 1:** Plasma total bilirubin and albumin levels. ND, not determined as untreated mutant mice do not survive up to P30.

Treatment	Age	TB (mg/dL)		Plasma albumin (g/L)	
		WT	MUT	WT	MUT
P0-P10 PT	P15	0.2 ± 0.1 (20)	14.9 ± 1.6 (21)***	16.2 ± 1.8 (20)	15.7 ± 2.1 (21) ns
HSA 2.5 g/kg/24h	P15	0.6 ± 0.1 (5)	28.8 ± 2.6 (7)***	22.9 ± 0.5 (5)	24.1 ± 1.4 (7) ns
HSA 5.0 g/kg/48h	P15	0.5 ± 0.1 (4)	24.3 ± 2.6 (15)***	26.1 ± 1.4 (4)	26.4 ± 2.6 (15) ns
HSA 5.0 g/kg/24h	P15	0.7 ± 0.1 (5)	25.1 ± 2.8 (15)***	27.4 ± 2.9 (5)	28.9 ± 2.2 (15) ns
HSA 7.5 g/kg/24h	P15	1.7 (1)	29.4 ± 1.6 (3)	35.9 ± 0 (1)	35.3 ± 1.1 (3)
P0-P10 PT	P16	0.2 ± 0.1 (7)	19.3 ± 3.5 (6)***	16.2 ± 1.1 (7)	15.8 ± 2.8 (6) ns
HSA 5.0 g/kg/48h	P16	0.3 ± 0.2 (7)	27.7 ± 2.8 (14)***	24.2 ± 4.2 (7)	21.7 ± 4.1 (14) ns
HSA 5.0 g/kg/24h	P16	0.8 ± 0.2 (6)	31.2 ± 4.2 (7)***	29.1 ± 4.4 (6)	29.5 ± 4.6 (7) ns
Untreated	P30	0.1 ± 0.1 (4)	ND	18.3 ± 1.8 (4)	ND
HSA 5.0 g/kg/24h	P30	0.1 ± 0.1 (5)	8.2 ± 1.2 (7)***	19.2 ± 1.5 (7)	16.7 ± 2.0 (7) ns
HSA 7.5 g/kg/24h	P30	0.1 ± 0.1 (5)	7.4 ± 1.05 (6)***	18.7 ± 2.3 (6)	17.9 ± 1.7 (6) ns

The number of animals is indicated between parenthesis. *** indicates a *p* < 0.001 (*t*-test between WT and treated mutant mice, within each treatment).
