# Supplementary material for: Albumin administration prevents neurological damage and death in a mouse model of severe neonatal hyperbilirubinemia
Source: Sci Rep. 2015 Nov 6;5:16203. doi: 10.1038/srep16203 (PMC4635426; doi:10.1038/srep16203)
Supplement: Supplementary Information [file srep16203-s1.pdf]

Supplementary data to:

Albumin administration prevents neurological damage and death in a mouse model of severe neonatal hyperbilirubinemia.

Simone Vodret[1], Giulia Bortolussi[1], Andrea B. Schreuder[2], Jana Jašprová[3], Libor Vitek[3], Henkjan J. Verkade[2] and Andrés F. Muro[1]

[1] International Centre for Genetic Engineering and Biotechnology (ICGEB), Trieste, Italy;

[2] Pediatric Gastroenterology and Hepatology, Department of Pediatrics, Center for Liver, Digestive, and Metabolic Diseases, University of Groningen, Beatrix Children's Hospital-University Medical Center, Groningen, the Netherlands

[3] Institute of Medical Biochemistry and Laboratory Diagnostics, 1st Faculty of Medicine, Charles University in Prague, Czech Republic

Supplementary Materials and Methods

Biochemical analyses of plasma samples

Blood samples were collected at different time points in mutant and WT littermates by cardiac puncture in EDTA-collecting tubes, at the moment of sacrificing the animals, as previously described [1]. Total bilirubin (TB) determination in plasma was performed using Direct and Total Bilirubin Reagent kit (BQ Kits, San Diego, CA), as described [2]. Absorbance values at 560 nm were obtained by using a multiplate reader (Perkin Elmer Envision Plate Reader, Walthman, MA). Bf was determined using a Zone Fluidics system (Global Flopro, Global Fia Inc, WA), as previously described by Ahlfors [3]. Albumin determination in plasma samples was performed with the Bromocresol Green method, adapting the method to use minimal volumes (2 µl of plasma), as previously described [2]. In each test a standard curve was performed by dilution of a stock solution (10 mg/ml) of human albumin (Albuman®; 200 g/L, Sanquin, Amsterdam, The Netherlands) in water. Absorbance values at 630 nm were obtained by using a multiplate reader (Perkin Elmer Envision Plate Reader, Walthman, MA).

Plasma alanine aminotransferase (ALT) and aspartate aminotransferase (AST) activity were determined using a Diagnostic ALT and AST test kit from Sigma-Aldrich, according to manufacturer's instructions (Cat. No MAK052, MAK055, St. Luis, MO).

Tissue bilirubin analysis

Tissues for bilirubin content determination (forebrain and cerebellum) were collected as previously described [2]. In particular, tissue bilirubin content was determined using HPLC with diode array detector (Agilent, Santa Clara, CA) as described previously by Zelenka [4]. Briefly, 300 pmol of mesobilirubin in DMSO (used as an internal standard) was added and samples were homogenized on ice by gkass rod. Bile pigments were then extracted into methanol/chloroform/hexane (10:5:1) solution at pH 6.0, and subsequently extracted in a minimum volume of hexane/carbonate buffer (pH 10) to remove contaminants. The resulting polar droplet (extract) was loaded onto C-8 reverse phase column (Phenomenex, Torrance, CA) and separated pigments were detected at 440 nm. The concentration of bilirubin was expressed as nmol/g of wet tissue weight. All steps were performed under dim light in aluminum-wrapped tubes.

Brain histology

Brains from each treatment and genotype were fixed with 4% PFA in PBS overnight at 4°C. After cryoprotection in 20% sucrose, 0.02% sodium azide in PBS, specimens were frozen in cryostat embedding medium (Bio-optica) and 14-μm sagittal sections were obtained in a cryostat. Nissl staining was performed as previously described [1,5]. For immunofluorescence, 14-μm sagittal sections were blocked for 2 hours at room temperature (RT) with 2.5% BSA in PBS 0.3% Triton X-100. After blocking, specimens were incubated with the primary antibody for 2 hours at RT in blocking solution with anti-calbindin (Synaptic Systems, Goettingen, Germany). After 3×5-minute washes with blocking solution, specimens were incubated with secondary antibody (Alexa Fluor 488; Invitrogen Carlsbad, CA) for 2 hours at RT. Nuclei were visualized by addition of Hoechst (10 μg/ml, Invitrogen) for 5 minutes after secondary antibody solution. Nissl-stained slides were mounted in Eukitt (Fluka, St Louis, MO), whereas immunostained slides were mounted in Mowiol 4–88 (Sigma-Aldrich). Images were acquired on a Nikon Eclipse E-800 epifluorescent microscope with a charge-coupled device camera (DMX 1200F; Nikon Amstelveen, The Netherlands). Digital images were collected using ACT-1 (Nikon) software. Analysis of the layer thickness was performed on Nissl-stained sections by measuring the layer depth (μm) as described [5]. PCs number was calculated by counting calbindin-positive cells in vermis sections along the entire cerebellum perimeter and expressed as linear density (cell/mm) as previously described [5]

The study was performed in a double-blind fashion: the genotype of the animals and the treatment were unknown to the surgeon, while a different investigator analyzed the data. Measurements were averaged for each animal.

Legends to Supplementary Figures

Supplementary Figure 1. No evidence of toxicity as a consequence of HSA administration. (A) Weight curve of HSA 7.5 g/kg/24 h. Two-way ANOVA, not significant. **(B) and (C)** Markers of liver damage alanine amino-transferase (ALT) **(B)** and aspartate amino-transferase (AST) **(C)** were determined for WT (n=4), P0-P10 PT (n=3), HSA 5.0 g/kg/24 h (n=3), and HSA 7.5 g/kg/24 h (n=3) -treated mutant mice at P16. Values represent mean ± SD (U/L). One-way ANOVA, not significant.

Supplementary Figure 2. Physiological plasma bilirubin increase. TB analyzed at P15 and P16 in P0-P10 PT, HSA 5.0 g/kg/48 h, and HSA 5.0 g/kg/24 h-treated mutant mice. *t*-test $*p<0.05$, $**p<0.01$, $***p<0.001$. Number of animals per treatment/genotype are indicated in Figure 5A.

Supplementary Figure 3. Plasma albumin and bilirubin levels at P30. (A) Plasma albumin levels at P30 for untreated WT (n=4), HSA 5.0 g/kg/24 h-treated WT (n=6) and MUT (n=6), HSA 7.5 g/kg/24 h-treated WT (n=7) and MUT(n=7). One way ANOVA, not significant. **(B)** TB levels of HSA 5.0 g/kg/24 h-treated WT (n=6) and MUT(n=6) and HSA 7.5 g/kg/24 h-treated WT (n=7) and MUT(n=7).

Supplementary References

1. Bortolussi G, Baj G, Vodret S*, et al.* Age-dependent pattern of cerebellar susceptibility to bilirubin neurotoxicity in vivo in mice. *Disease models & mechanisms* 2014; **7**: 1057-1068.
2. Bortolussi G, Zentillin L, Vanikova J*, et al.* Life-Long Correction of Hyperbilirubinemia with a Neonatal Liver-Specific AAV-Mediated Gene Transfer in a Lethal Mouse Model of Crigler-Najjar Syndrome. *Human gene therapy* 2014; **25**: 844-855.
3. Ahlfors CE, Marshall GD, Wolcott DK*, et al.* Measurement of unbound bilirubin by the peroxidase test using Zone Fluidics. *Clinica chimica acta; international journal of clinical chemistry* 2006; **365**: 78-85.
4. Zelenka J, Lenicek M, Muchova L*, et al.* Highly sensitive method for quantitative determination of bilirubin in biological fluids and tissues. *Journal of chromatography B, Analytical technologies in the biomedical and life sciences* 2008; **867**: 37-42.
5. Bortolussi G, Zentilin L, Baj G*, et al.* Rescue of bilirubin-induced neonatal lethality in a mouse model of Crigler-Najjar syndrome type I by AAV9-mediated gene transfer. *FASEB journal : official publication of the Federation of American Societies for Experimental Biology* 2012; **26**: 1052-1063.

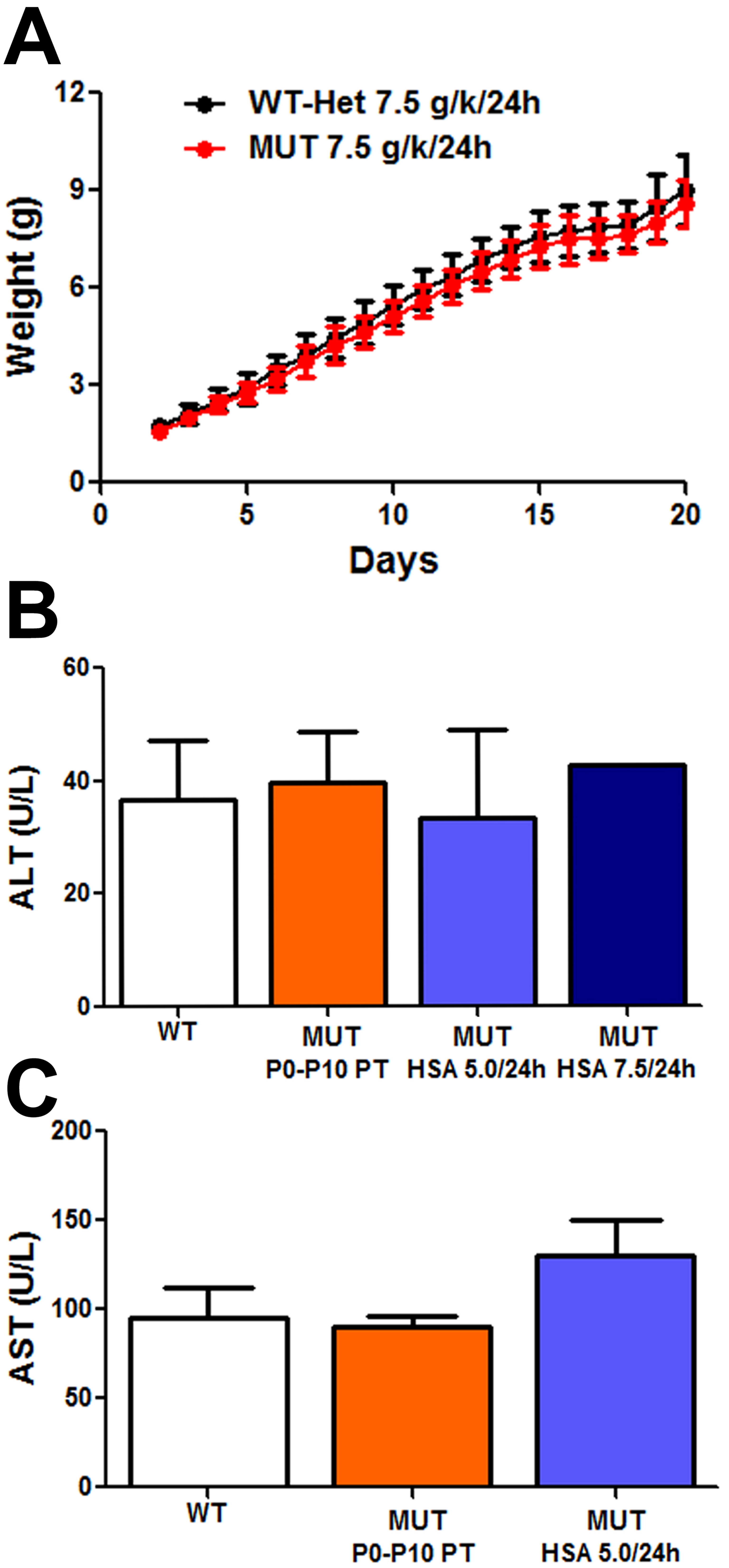
A
WT-Het 7.5 g/k/24h
MUT 7.5 g/k/24h
Weight (g)
12
9
6
3
0
0
5
10
15
20
Days
B
ALT (U/L)
60
40
20
0
WT
MUT
P0-P10 PT
MUT
HSA 5.0/24h
MUT
HSA 7.5/24h
C
AST (U/L)
200
150
100
50
0
WT
MUT
P0-P10 PT
MUT
HSA 5.0/24h

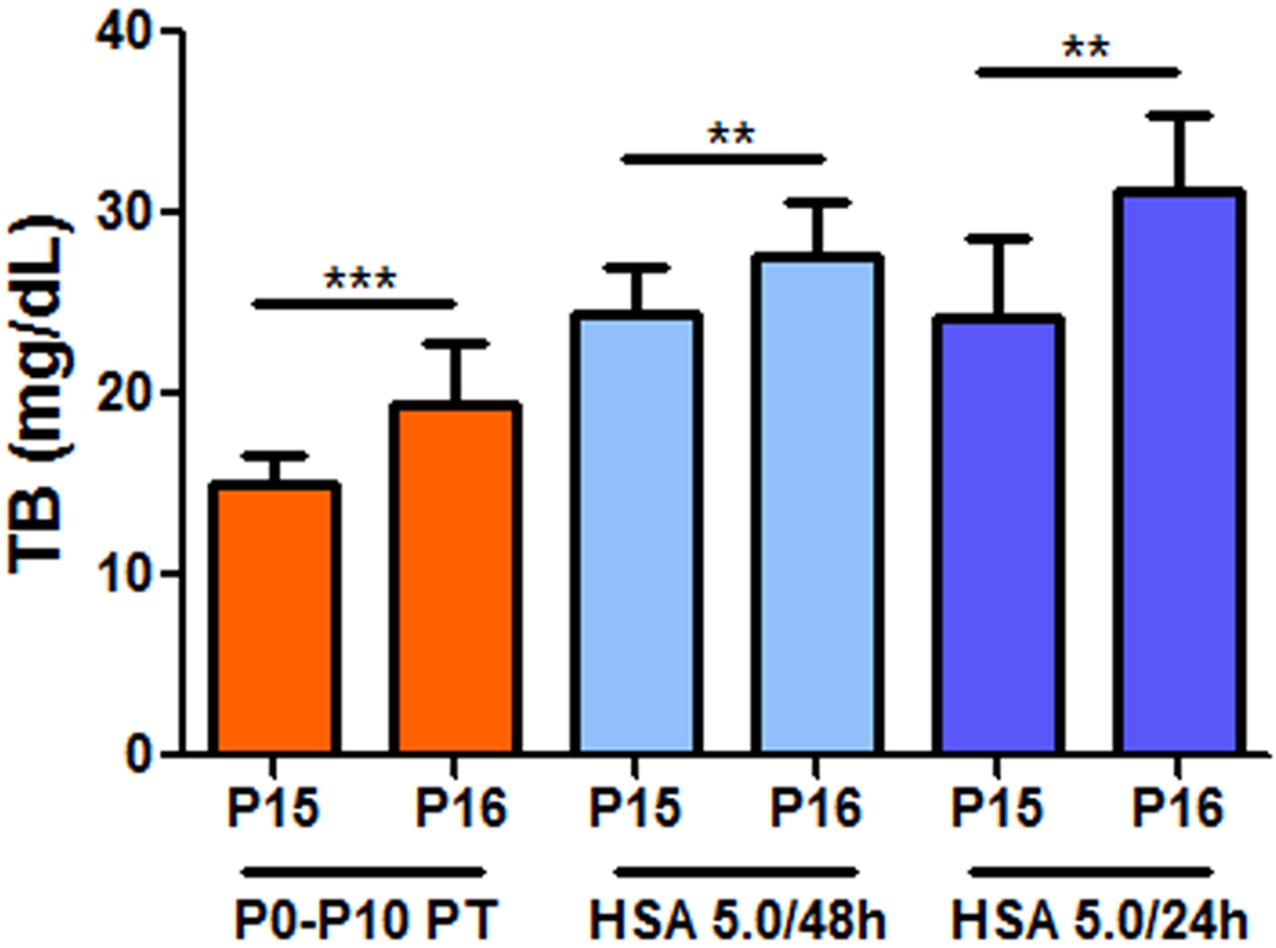
TB (mg/dL)
40
30
20
10
0
***
**
**
P15
P16
P15
P16
P15
P16
P0-P10 PT
HSA 5.0/48h
HSA 5.0/24h

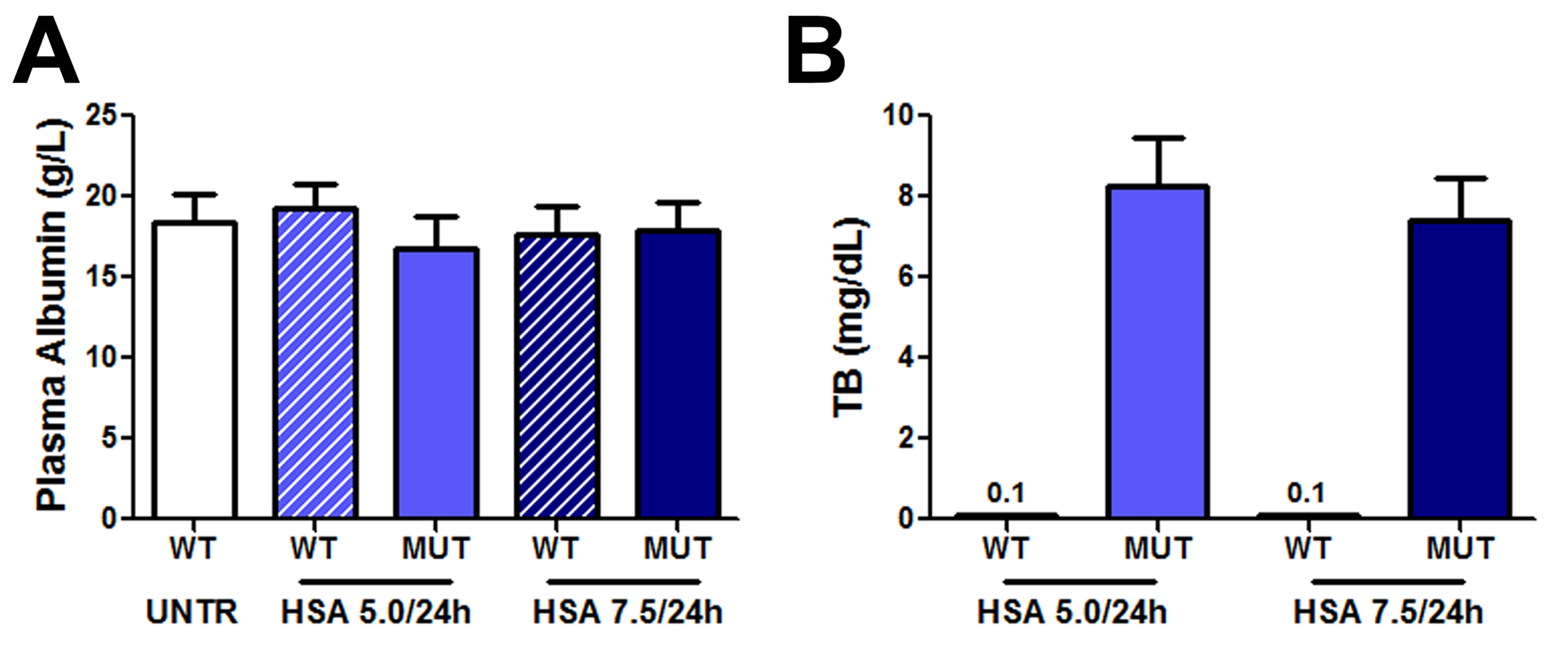
A
B
Plasma Albumin (g/L)
25
20
15
10
5
0
WT
WT
MUT
WT
MUT
UNTR
HSA 5.0/24h
HSA 7.5/24h
TB (mg/dL)
10
8
6
4
2
0
0.1
0.1
WT
MUT
WT
MUT
HSA 5.0/24h
HSA 7.5/24h
